# Crystal structure of 2-amino-5,6,7,8-tetra­hydro-7,7-dimethyl-4-(naphthalen-2-yl)-5-oxo-4*H*-chromene-3-carbo­nitrile

**DOI:** 10.1107/S2056989022005199

**Published:** 2022-05-20

**Authors:** Ali M. S. Hebishy, Galal H. Elgemeie, Rasha A. E. Ali, Peter G. Jones

**Affiliations:** a Chemistry Department, Faculty of Science, Helwan University, Cairo, Egypt; b Institut für Anorganische und Analytische Chemie, Technische Universität Braunschweig, Hagenring 30, D-38106 Braunschweig, Germany; University of Aberdeen, Scotland

**Keywords:** chromene, naphth­yl, crystal structure

## Abstract

In the title compound, C_22_H_20_N_2_O_2_, both six-membered rings of the fused heterocyclic system display envelope conformations; the two carbon atoms bearing the methyl groups and the naphthyl substituent both lie outside the planes of the other atoms of each ring. In the crystal, the amino group forms hydrogen bonds of the types N—H⋯O=C and N—H⋯N≡C, leading to the formation of a double layer structure propagating parallel to the *bc* plane. Weak C—H⋯O and C—H⋯π inter­actions may reinforce the layers.

## Abstract 2

Both six-membered rings of the fused heterocyclic system of the title compound display envelope conformations. Two hydrogen bonds involving the amino group lead to a double-layer structure.

## 1. Chemical context

Six-membered heterocycles involving 4*H*-pyran units represent an important class of biologically active synthetic and natural products, many of which attract the inter­est of the drug industry (Lega *et al.*, 2016 ▸). Pyrans possess anti­microbial (Dazmiri *et al.*, 2020 ▸), anti­tuberculosis (Kalaria *et al.*, 2014 ▸) and anti­tumor (Wang *et al.*, 2014 ▸) activities, whereby 4*H-*pyrans are moieties in a series of natural products (Singh *et al.*, 1996 ▸). A number of 4*H*-pyrans are used, for example, as photoactive ingredients (Armesto *et al.*, 1989 ▸) or agrochemicals (Kumar *et al.*, 2009 ▸). Synthetically, they are inter­mediates for the synthesis of heterocyclic compounds such as pyran­opyrimidines and pyran­opyrazoles (Elgemeie *et al.*, 1987 ▸, 1988 ▸) and consequently the synthesis of 4*H*-pyrans themselves is of inter­est to organic chemists.

Some time ago, we reported the synthesis of pyridine-2(1*H*)-thio­nes and their condensed derivatives from the reactions of aryl­methyl­ene­cyano­thio­acetamides with suitable active methyl­ene compounds (Elgemeie *et al.*, 2002 ▸).We also described the reaction of the dimedone **1** with naphthyl­methyl­ene­cyano­thio­acetamide to produce a condensed pyridine-2(1*H*)-thione (Attia *et al.*, 1997 ▸). The course of this reaction prompted us to investigate how **1** would react with naphthyl­methyl­ene­cyano­acetamide [2-cyano-3-(naphthalen-2-yl)acryl­amide, **2**] in boiling ethanol containing tri­ethyl­amine. The product was shown to be neither of the expected condensed pyridin-2(1*H*)-ones **3** or **4** but rather the condensed pyran nitrile **5** (Scheme 1). The latter structure was inferred on the basis of elemental analysis and spectroscopic data: thus, the mass spectrum of **5** was compatible with the mol­ecular formula C_22_H_20_N_2_O_2_ (*M* ^+^, 344), and the ^1^H NMR spectrum had signals at 4.37 (pyran-4*H*), 7.06 (*br*, NH_2_) and 7.29–7.90 (*m*, ArH). 
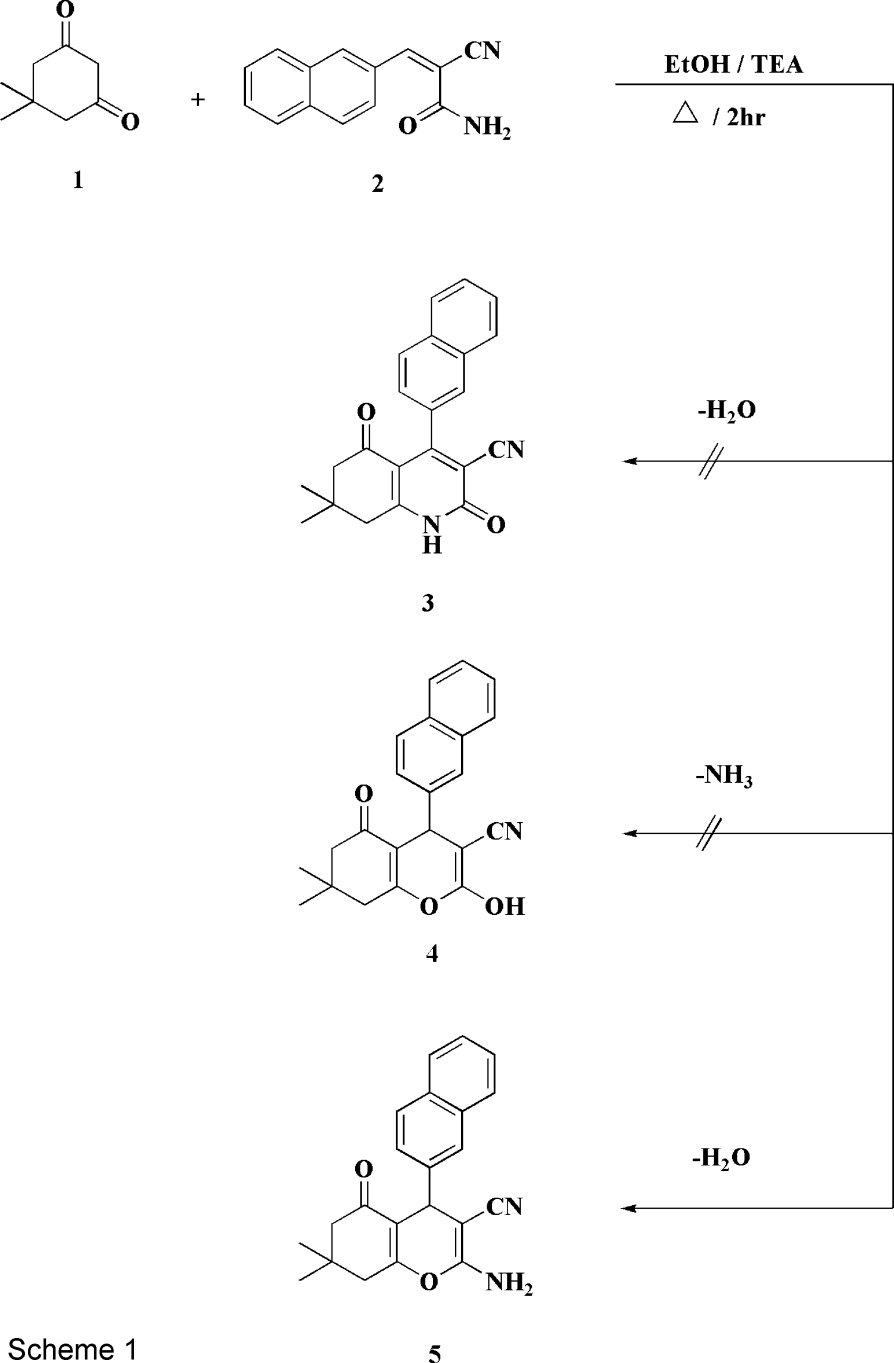


We assume that the formation of **5** proceeds *via* addition of the active methyl­ene group of **1** to the double bond of **2** to give the inter­mediates **6**, **7** and then **8**, the latter finally losing one mol­ecule of water to give the final product **5** (Scheme 2). In order to establish the structure of this compound unambiguously, its crystal structure was determined and is reported here. 
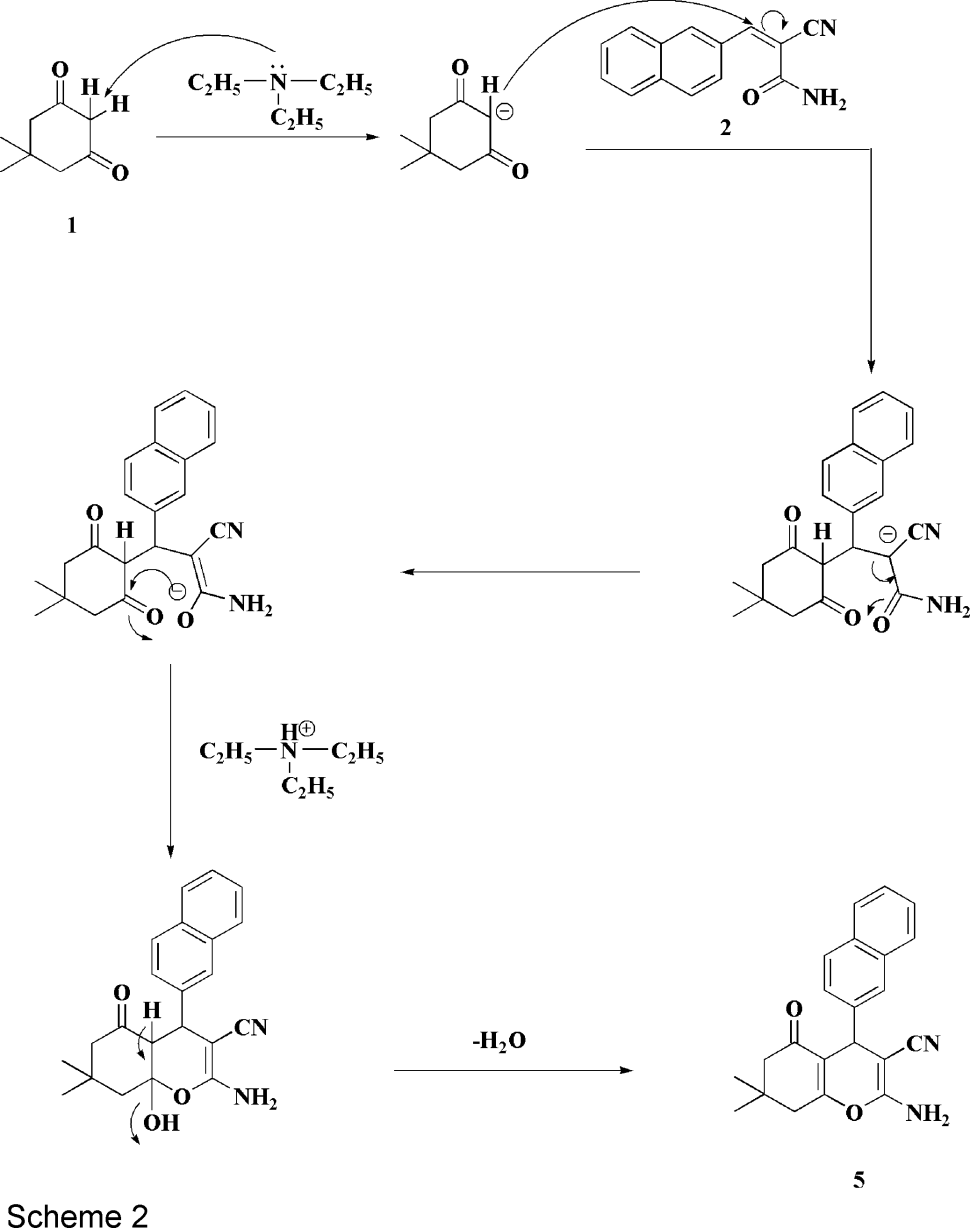


## 2. Structural commentary

**Figure 1:**
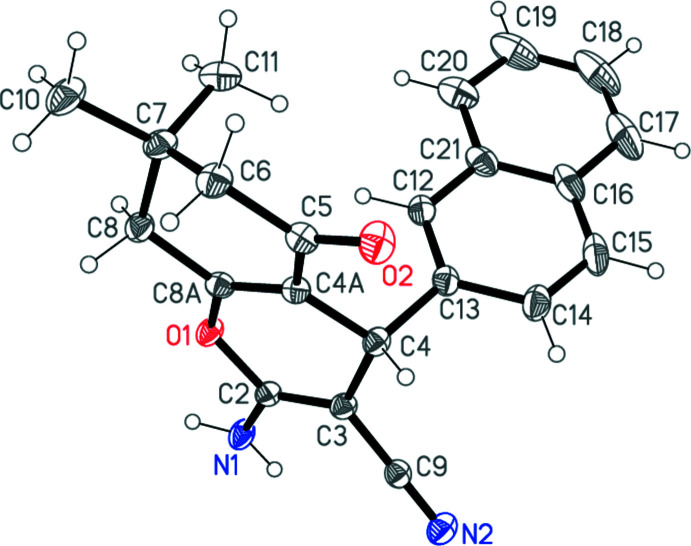
The mol­ecular structure of **5** in the crystal. Ellipsoids represent 50% probability levels.

The mol­ecular structure of **5** is shown in Fig. 1 ▸ and it confirms the postulated structure noted above. Both six-membered rings display envelope conformations in which five atoms are reasonably coplanar (for torsion angles see Table 1 ▸): C4 deviates by 0.317 (1) Å from the mean plane (I) of atoms O1/C2/C3/C4*A*/C8*A* (r.m.s. deviation = 0.031 Å), and C7 lies 0.653 (2) Å outside the mean plane (II) of C4*A*/C5–C8 (r.m.s. deviation = 0.030 Å). The inter­planar angle I/II is 9.97 (4)°. The naphthyl ring system (r.m.s. deviation = 0.012 Å) is effectively perpendicular to plane I [inter­planar angle = 86.56 (3)°]. The amino group is almost planar (r.m.s. deviation of C2/N1/H01/H02 = 0.01 Å) and deviates slightly from plane I [inter­planar angle = 10.0 (6)°].

## 3. Supra­molecular features

**Figure 2:**
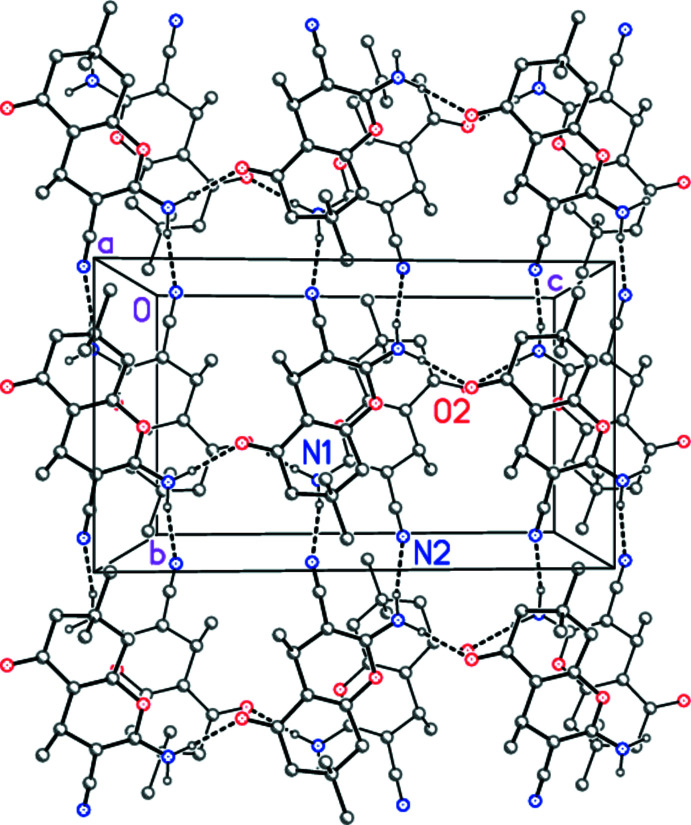
Crystal packing of **5** viewed parallel to the *a* axis in the region *x* ≃ 0.5. Dashed lines indicate classical hydrogen bonds. Naphthyl rings are reduced to the *ipso* carbon atoms for clarity. Hydrogen atoms not involved in classical hydrogen bonding are omitted. The figure is depth-coded; mol­ecules of the lower layer are drawn with thinner bonds. Atom labels indicate the asymmetric unit (which lies in the lower layer).

In the crystal, the amino group acts as donor for two classical hydrogen bonds (Table 2 ▸). This leads to a double layer structure (Fig. 2 ▸) propagating parallel to the *bc* plane. The H⋯O separation of the weak hydrogen bond C6—H6*B*⋯N2 (*x*, −1 + *y*, *z*) is rather long at 2.69 Å but acceptably linear (160°) and presumably reinforces the layer structure, but is not shown in Fig. 2 ▸. The short contact C10—H10*B*⋯*Cg* (C12–16/C21), with H⋯*Cg* 2.79 Å and a C—H⋯*Cg* angle of 139°, may represent a C—H⋯π inter­action between the double layers. There are no short π–π stacking contacts.

## 4. Database survey

**Figure 3:**
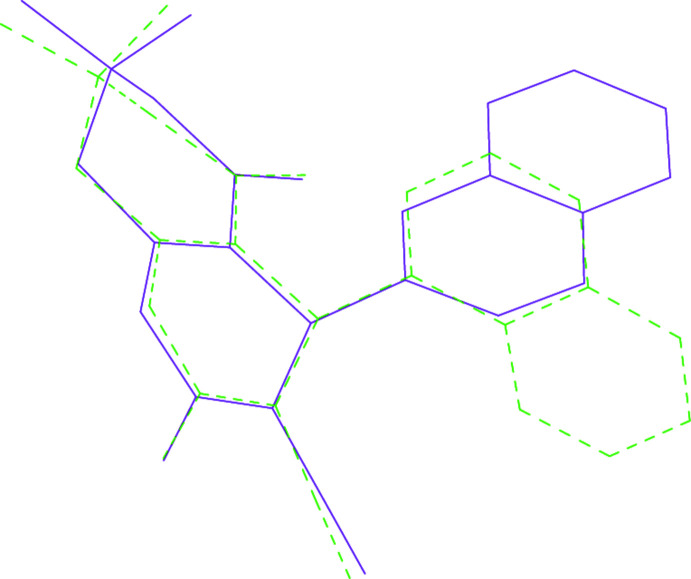
A least-squares fit of **5** (violet, full bonds) to ETOKIH (Nesterov *et al.*, 2004 ▸; green, dashed bonds). Hydrogen atoms were not considered.

A search of the Cambridge Database (Version 2021.3.0; Groom *et al.*, 2016 ▸) showed that the motif of a 4-substituted 2-amino-5,6,7,8-tetra­hydro-7,7-dimethyl-5-oxo-4*H*-chromene-3-carbo­nitrile has been the subject of many structure determinations. A total of 54 hits with variously substituted phenyl groups was found, which reduces to 32 when duplicate structure determinations, various solvates and polymorphs are not considered. For all but one of these structures, the 4-position also bears a hydrogen atom, the exception being the 4-methyl, 4-nitro­phenyl derivative (Cai *et al.*, 2012 ▸; refcode TESNEM). Additionally, the 4-(1-naphth­yl) derivative was found (Nesterov *et al.*, 2004 ▸; refcode ETOKIH), which is an isomer of the title compound **5**. The packing of ETOKIH is quite different from that of **5**; the hydrogen atom corresponding to H01 in **5** forms N—H⋯N hydrogen bonds, leading to inversion dimers, whereas the other NH hydrogen atom is not involved in hydrogen bonding. A least-squares overlay of **5** and ETOKIH (excluding methyl groups and all naphthyl carbon atoms except the *ipso* C atom) gave an r.m.s. deviation of 0.15 Å; Fig. 3 ▸ shows the slight differences in ring conformation.

## 5. Synthesis and crystallization

A mixture of dimedone **1** (0.010 mol), 2-cyano-3-(naphthalen-2-yl)acryl­amide **2** (0.010 mol) and tri­ethyl­amine (0.010 mol) in ethanol (10 ml) was refluxed for 2 h. The solid precipitate that formed was filtered off and recrystallized from ethanol solution to give pale yellow crystals of **5** in 90% yield, m.p. 474–475 K; IR (KBr, cm^−1^): υ 3345, 3258 (NH_2_), 2188 (CN), 1683 (C=O). ^1^H NMR (400 MHz DMSO-*d*_6_) *δ*: 1.11 (*s*, 3H, CH_3_), 1.53 (*s*, 3H, CH_3_), 2.07 (*d*, 2H, CH_2_), 2.14 (*d*, 2H, CH_2_), 4.37 (*s*, 1H, CH-pyran), 7.06 (*s*, *br*, 2H, NH_2_), 7.29–7.90 (*m*, 7H, C_10_H_7_). ^13^C NMR (100 MHz, DMSO-*d_6_*) *δ*: 27.2, 28.8, 32.2, 36.3, 50.4, 58.6 (aliphatic C), 120.2 (CN), 113.0, 142.2, 158.9, 163.0 (ethyl­ene C), 120.2-133.3 (aromatic C), 196.2 (C=O). MS (EI): *m*/*z* 344 [*M* ^+^]. Analysis calculated for C_22_H_20_N_2_O_2_: C 76.72; H 5.85; N 8.13%. Found: C 76.6; H 5.7; N 8.1%.

## 6. Refinement

Crystal data, data collection and structure refinement details are summarized in Table 3 ▸. The hydrogen atoms of the NH_2_ group were refined freely, but with N—H distances restrained to be approximately equal using a SADI instruction in *SHELXL*. The methyl groups were included as idealised rigid groups allowed to rotate but not tip (C—H = 0.98 Å; H—C—H = 109.5°). The other hydrogen atoms were included using a riding model starting from calculated positions (C—H = 0.95, 0.98 and 1.00 Å for aromatic, methyl­ene and methine H atoms, respectively). The *U*_iso_(H) values were fixed at 1.5 × *U*_eq_ of the parent carbon atoms for the methyl groups and 1.2 × *U*_eq_ for other hydrogen atoms.

## Supplementary Material

Crystal structure: contains datablock(s) I, global. DOI: 10.1107/S2056989022005199/hb8021sup1.cif

Structure factors: contains datablock(s) I. DOI: 10.1107/S2056989022005199/hb8021Isup2.hkl

CCDC reference: 2172530

Additional supporting information:  crystallographic information; 3D view; checkCIF report

## Figures and Tables

**Table 1:** Selected torsion angles (°)

C8*A*—O1—C2—C3	−8.36 (13)	C5—C6—C7—C8	54.40 (11)
O1—C2—C3—C4	−9.65 (14)	C6—C7—C8—C8*A*	−47.78 (11)
C2—C3—C4—C4*A*	22.65 (13)	C4—C4*A*—C8*A*—O1	5.99 (15)
C3—C4—C4*A*—C8*A*	−20.86 (12)	C5—C4*A*—C8*A*—C8	5.73 (15)
C8*A*—C4*A*—C5—C6	0.61 (14)	C2—O1—C8*A*—C4*A*	10.40 (13)
C4*A*—C5—C6—C7	−31.83 (13)	C7—C8—C8*A*—C4*A*	19.52 (14)

**Table 2:** Hydrogen-bond geometry (Å, °)

*D*—H⋯*A*	*D*—H	H⋯*A*	*D*⋯*A*	*D*—H⋯*A*
N1—H01⋯N2^i^	0.90 (1)	2.11 (1)	2.9948 (12)	170 (1)
N1—H02⋯O2^ii^	0.90 (1)	1.94 (1)	2.8404 (11)	176 (1)
C6—H6*B*⋯N2^iii^	0.99	2.69	3.6366 (14)	160

Symmetry codes: (i) 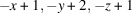 ; (ii) 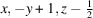 ; (iii) 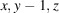 .

**Table 3:** Experimental details

Crystal data	
Chemical formula	C_22_H_20_N_2_O_2_
*M*_r_	344.40
Crystal system, space group	Monoclinic, *C*2/*c*
Temperature (K)	100
*a*, *b*, *c* (Å)	25.3144 (3), 9.25765 (11), 15.6778 (2)
β (°)	97.8724 (10)
*V* (Å^3^)	3639.51 (8)
*Z*	8
Radiation type	Cu *K*α
μ (mm^−1^)	0.65
Crystal size (mm)	0.08 × 0.05 × 0.02

Data collection	
Diffractometer	XtaLAB Synergy, HyPix
Absorption correction	Multi-scan (*CrysAlis PRO*; Rigaku OD, 2021 ▸)
*T*_min_, *T*_max_	0.826, 1.000
No. of measured, independent and observed [*I* > 2σ(*I*)] reflections	61093, 3856, 3694
*R*_int_	0.030
(sin θ/λ)_max_ (Å^−1^)	0.634

Refinement	
*R*[*F* ^2^ > 2σ(*F* ^2^)], *wR*(*F* ^2^), *S*	0.036, 0.086, 1.07
No. of reflections	3856
No. of parameters	245
No. of restraints	1
H-atom treatment	H atoms treated by a mixture of independent and constrained refinement
Δρ_max_, Δρ_min_ (e Å^−3^)	0.22, −0.20

Computer programs: *CrysAlis PRO* (Rigaku OD, 2021 ▸), *SHELXT* (Sheldrick, 2015*a* ▸), *SHELXL2018/3* (Sheldrick, 2015*b* ▸) and *XP* (Siemens, 1994 ▸).

## supplementary crystallographic information

### Crystal data

**Table:**

C_22_H_20_N_2_O_2_	*F*(000) = 1456
*M**_r_* = 344.40	*D*_x_ = 1.257 Mg m^−^^3^
Monoclinic, *C*2/*c*	Cu *K*α radiation, λ = 1.54184 Å
*a* = 25.3144 (3) Å	Cell parameters from 35871 reflections
*b* = 9.25765 (11) Å	θ = 3.5–77.4°
*c* = 15.6778 (2) Å	µ = 0.65 mm^−^^1^
β = 97.8724 (10)°	*T* = 100 K
*V* = 3639.51 (8) Å^3^	Lath, colourless
*Z* = 8	0.08 × 0.05 × 0.02 mm

### Data collection

**Table:**

XtaLAB Synergy, HyPix diffractometer	3856 independent reflections
Radiation source: micro-focus sealed X-ray tube	3694 reflections with *I* > 2σ(*I*)
Detector resolution: 10.0000 pixels mm^-1^	*R*_int_ = 0.030
ω scans	θ_max_ = 77.6°, θ_min_ = 3.5°
Absorption correction: multi-scan (CrysalisPro; Rigaku OD, 2021)	*h* = −31→31
*T*_min_ = 0.826, *T*_max_ = 1.000	*k* = −11→11
61093 measured reflections	*l* = −19→19

### Refinement

**Table:**

Refinement on *F*^2^	Primary atom site location: dual
Least-squares matrix: full	Hydrogen site location: mixed
*R*[*F*^2^ > 2σ(*F*^2^)] = 0.036	H atoms treated by a mixture of independent and constrained refinement
*wR*(*F*^2^) = 0.086	*w* = 1/[σ^2^(*F*_o_^2^) + (0.0362*P*)^2^ + 2.6486*P*] where *P* = (*F*_o_^2^ + 2*F*_c_^2^)/3
*S* = 1.07	(Δ/σ)_max_ < 0.001
3856 reflections	Δρ_max_ = 0.22 e Å^−^^3^
245 parameters	Δρ_min_ = −0.20 e Å^−^^3^
1 restraint	

### Special details

**Table:**

Geometry. All esds (except the esd in the dihedral angle between two l.s. planes) are estimated using the full covariance matrix. The cell esds are taken into account individually in the estimation of esds in distances, angles and torsion angles; correlations between esds in cell parameters are only used when they are defined by crystal symmetry. An approximate (isotropic) treatment of cell esds is used for estimating esds involving l.s. planes. Least-squares planes (x,y,z in crystal coordinates) and deviations from them (* indicates atom used to define plane) 24.6913 (0.0028) x - 2.0266 (0.0045) y - 2.5063 (0.0065) z = 7.9737 (0.0050) * -0.0099 (0.0006) C4A * -0.0265 (0.0007) C5 * 0.0300 (0.0005) C6 * -0.0353 (0.0005) C8 * 0.0417 (0.0007) C8A -0.6525 (0.0015) C7 Rms deviation of fitted atoms = 0.0306 23.4020 (0.0039) x - 3.5289 (0.0034) y - 1.8463 (0.0073) z = 7.1162 (0.0054) Angle to previous plane (with approximate esd) = 9.967 ( 0.036 ) * -0.0423 (0.0006) O1 * 0.0228 (0.0006) C2 * -0.0021 (0.0005) C3 * -0.0200 (0.0005) C4A * 0.0415 (0.0006) C8A -0.3166 (0.0014) C4 Rms deviation of fitted atoms = 0.0298 - 6.0033 (0.0056) x - 6.6409 (0.0023) y + 10.6832 (0.0042) z = 0.1010 (0.0050) Angle to previous plane (with approximate esd) = 86.556 ( 0.027 ) * -0.0188 (0.0008) C12 * -0.0048 (0.0008) C13 * 0.0149 (0.0009) C14 * 0.0098 (0.0010) C15 * -0.0015 (0.0011) C16 * -0.0151 (0.0011) C17 * -0.0093 (0.0011) C18 * 0.0169 (0.0011) C19 * 0.0111 (0.0010) C20 * -0.0032 (0.0010) C21 Rms deviation of fitted atoms = 0.0119

### Fractional atomic coordinates and isotropic or equivalent isotropic displacement parameters (Å^2^)

**Table:**

	*x*	*y*	*z*	*U*_iso_*/*U*_eq_	
O1	0.42046 (3)	0.54277 (7)	0.46059 (4)	0.01878 (16)	
C2	0.44459 (4)	0.67304 (10)	0.48220 (6)	0.01719 (19)	
N1	0.45324 (4)	0.74447 (10)	0.41141 (6)	0.02197 (19)	
H01	0.4646 (6)	0.8361 (15)	0.4146 (9)	0.038 (4)*	
H02	0.4446 (6)	0.7028 (15)	0.3594 (8)	0.036 (4)*	
C3	0.45648 (4)	0.71509 (10)	0.56594 (6)	0.0174 (2)	
C4	0.43643 (4)	0.63303 (11)	0.63906 (6)	0.0175 (2)	
H4	0.465617	0.629568	0.688927	0.021*	
C4A	0.42383 (4)	0.48120 (10)	0.60884 (6)	0.0172 (2)	
C5	0.42044 (4)	0.36809 (11)	0.67350 (6)	0.0198 (2)	
O2	0.42982 (3)	0.39810 (8)	0.75024 (5)	0.02716 (18)	
C6	0.40710 (4)	0.21643 (11)	0.64222 (7)	0.0231 (2)	
H6A	0.389602	0.164811	0.686041	0.028*	
H6B	0.440604	0.164729	0.636056	0.028*	
C7	0.37033 (4)	0.21205 (11)	0.55576 (7)	0.0223 (2)	
C8	0.39608 (4)	0.30239 (11)	0.49014 (6)	0.0204 (2)	
H8A	0.426832	0.248784	0.473153	0.025*	
H8B	0.369856	0.316614	0.437883	0.025*	
C8A	0.41454 (4)	0.44589 (10)	0.52524 (6)	0.01697 (19)	
C9	0.48381 (4)	0.84661 (11)	0.58446 (6)	0.0183 (2)	
N2	0.50703 (4)	0.95261 (10)	0.60116 (6)	0.0238 (2)	
C10	0.36359 (5)	0.05655 (12)	0.52270 (8)	0.0311 (3)	
H10A	0.338967	0.055087	0.468724	0.047*	
H10B	0.349210	−0.003296	0.565660	0.047*	
H10C	0.398300	0.018361	0.512555	0.047*	
C11	0.31556 (4)	0.27340 (14)	0.56747 (8)	0.0312 (3)	
H11A	0.320032	0.368819	0.594563	0.047*	
H11B	0.298005	0.208440	0.604174	0.047*	
H11C	0.293616	0.282185	0.511149	0.047*	
C12	0.33761 (4)	0.68876 (11)	0.62556 (6)	0.0203 (2)	
H12	0.332117	0.622111	0.579111	0.024*	
C13	0.38823 (4)	0.70882 (11)	0.66778 (6)	0.0189 (2)	
C14	0.39613 (5)	0.80926 (12)	0.73650 (7)	0.0265 (2)	
H14	0.430947	0.823540	0.766542	0.032*	
C15	0.35431 (5)	0.88596 (13)	0.76020 (7)	0.0330 (3)	
H15	0.360637	0.953461	0.806098	0.040*	
C16	0.30184 (5)	0.86665 (13)	0.71766 (7)	0.0308 (3)	
C17	0.25720 (6)	0.94476 (16)	0.73986 (9)	0.0462 (4)	
H17	0.262423	1.014310	0.784776	0.055*	
C18	0.20725 (6)	0.92164 (18)	0.69796 (10)	0.0517 (4)	
H18	0.178037	0.975221	0.713572	0.062*	
C19	0.19874 (5)	0.81873 (17)	0.63165 (10)	0.0460 (4)	
H19	0.163632	0.801759	0.603416	0.055*	
C20	0.24071 (5)	0.74252 (14)	0.60733 (8)	0.0336 (3)	
H20	0.234466	0.674176	0.561864	0.040*	
C21	0.29329 (4)	0.76481 (12)	0.64938 (7)	0.0249 (2)	

### Atomic displacement parameters (Å^2^)

**Table:**

	*U*^11^	*U*^22^	*U*^33^	*U*^12^	*U*^13^	*U*^23^
O1	0.0252 (4)	0.0155 (3)	0.0155 (3)	−0.0050 (3)	0.0022 (3)	0.0003 (3)
C2	0.0174 (4)	0.0155 (4)	0.0189 (5)	−0.0018 (3)	0.0030 (3)	−0.0002 (4)
N1	0.0322 (5)	0.0182 (4)	0.0159 (4)	−0.0071 (4)	0.0049 (3)	−0.0012 (3)
C3	0.0179 (4)	0.0165 (5)	0.0176 (5)	−0.0017 (4)	0.0024 (3)	0.0004 (4)
C4	0.0204 (5)	0.0176 (5)	0.0141 (4)	−0.0018 (4)	0.0005 (3)	0.0008 (4)
C4A	0.0162 (4)	0.0164 (5)	0.0190 (5)	0.0003 (3)	0.0025 (3)	0.0014 (4)
C5	0.0195 (5)	0.0199 (5)	0.0204 (5)	0.0019 (4)	0.0036 (4)	0.0030 (4)
O2	0.0386 (4)	0.0249 (4)	0.0177 (4)	0.0001 (3)	0.0028 (3)	0.0039 (3)
C6	0.0281 (5)	0.0175 (5)	0.0238 (5)	−0.0008 (4)	0.0038 (4)	0.0050 (4)
C7	0.0246 (5)	0.0179 (5)	0.0247 (5)	−0.0042 (4)	0.0042 (4)	0.0025 (4)
C8	0.0238 (5)	0.0171 (5)	0.0207 (5)	−0.0023 (4)	0.0042 (4)	−0.0006 (4)
C8A	0.0163 (4)	0.0160 (4)	0.0189 (5)	0.0003 (3)	0.0036 (3)	0.0030 (4)
C9	0.0196 (5)	0.0203 (5)	0.0148 (4)	0.0007 (4)	0.0023 (3)	0.0008 (4)
N2	0.0290 (5)	0.0208 (4)	0.0210 (4)	−0.0049 (4)	0.0018 (3)	−0.0004 (3)
C10	0.0423 (7)	0.0197 (5)	0.0308 (6)	−0.0092 (5)	0.0036 (5)	0.0022 (4)
C11	0.0219 (5)	0.0343 (6)	0.0380 (6)	−0.0061 (5)	0.0062 (5)	0.0034 (5)
C12	0.0256 (5)	0.0172 (5)	0.0188 (5)	0.0001 (4)	0.0054 (4)	0.0007 (4)
C13	0.0265 (5)	0.0162 (5)	0.0148 (4)	−0.0013 (4)	0.0057 (4)	0.0018 (4)
C14	0.0361 (6)	0.0251 (5)	0.0183 (5)	−0.0046 (4)	0.0041 (4)	−0.0030 (4)
C15	0.0524 (7)	0.0264 (6)	0.0228 (5)	0.0005 (5)	0.0143 (5)	−0.0063 (4)
C16	0.0433 (7)	0.0259 (6)	0.0274 (6)	0.0071 (5)	0.0193 (5)	0.0043 (5)
C17	0.0621 (9)	0.0411 (8)	0.0421 (7)	0.0186 (7)	0.0304 (7)	0.0038 (6)
C18	0.0496 (8)	0.0577 (9)	0.0551 (9)	0.0287 (7)	0.0329 (7)	0.0181 (7)
C19	0.0308 (7)	0.0577 (9)	0.0527 (8)	0.0146 (6)	0.0174 (6)	0.0211 (7)
C20	0.0270 (6)	0.0363 (6)	0.0391 (7)	0.0045 (5)	0.0098 (5)	0.0102 (5)
C21	0.0291 (5)	0.0219 (5)	0.0261 (5)	0.0029 (4)	0.0121 (4)	0.0071 (4)

### Geometric parameters (Å, º)

**Table:**

O1—C2	1.3733 (11)	C17—C18	1.359 (2)
O1—C8A	1.3769 (11)	C18—C19	1.405 (2)
C2—N1	1.3355 (13)	C19—C20	1.3724 (18)
C2—C3	1.3628 (13)	C20—C21	1.4172 (17)
C3—C9	1.4109 (13)	N1—H01	0.895 (13)
C3—C4	1.5193 (13)	N1—H02	0.901 (13)
C4—C4A	1.5035 (13)	C4—H4	1.0000
C4—C13	1.5278 (14)	C6—H6A	0.9900
C4A—C8A	1.3400 (14)	C6—H6B	0.9900
C4A—C5	1.4681 (13)	C8—H8A	0.9900
C5—O2	1.2259 (13)	C8—H8B	0.9900
C5—C6	1.5101 (14)	C10—H10A	0.9800
C6—C7	1.5362 (15)	C10—H10B	0.9800
C7—C10	1.5316 (15)	C10—H10C	0.9800
C7—C11	1.5323 (15)	C11—H11A	0.9800
C7—C8	1.5384 (14)	C11—H11B	0.9800
C8—C8A	1.4885 (13)	C11—H11C	0.9800
C9—N2	1.1553 (13)	C12—H12	0.9500
C12—C13	1.3721 (14)	C14—H14	0.9500
C12—C21	1.4173 (14)	C15—H15	0.9500
C13—C14	1.4164 (14)	C17—H17	0.9500
C14—C15	1.3678 (17)	C18—H18	0.9500
C15—C16	1.4135 (18)	C19—H19	0.9500
C16—C21	1.4206 (17)	C20—H20	0.9500
C16—C17	1.4244 (17)		
			
C2—O1—C8A	118.69 (7)	C12—C21—C16	118.93 (10)
N1—C2—C3	128.28 (9)	C2—N1—H01	120.8 (9)
N1—C2—O1	110.35 (8)	C2—N1—H02	119.5 (9)
C3—C2—O1	121.37 (9)	H01—N1—H02	119.5 (13)
C2—C3—C9	118.82 (9)	C4A—C4—H4	108.6
C2—C3—C4	122.11 (9)	C3—C4—H4	108.6
C9—C3—C4	118.77 (8)	C13—C4—H4	108.6
C4A—C4—C3	107.94 (8)	C5—C6—H6A	109.0
C4A—C4—C13	112.25 (8)	C7—C6—H6A	109.0
C3—C4—C13	110.83 (8)	C5—C6—H6B	109.0
C8A—C4A—C5	118.84 (9)	C7—C6—H6B	109.0
C8A—C4A—C4	122.52 (9)	H6A—C6—H6B	107.8
C5—C4A—C4	118.63 (8)	C8A—C8—H8A	109.2
O2—C5—C4A	119.62 (9)	C7—C8—H8A	109.2
O2—C5—C6	122.28 (9)	C8A—C8—H8B	109.2
C4A—C5—C6	118.07 (9)	C7—C8—H8B	109.2
C5—C6—C7	113.12 (8)	H8A—C8—H8B	107.9
C10—C7—C11	109.17 (9)	C7—C10—H10A	109.5
C10—C7—C6	110.45 (9)	C7—C10—H10B	109.5
C11—C7—C6	109.48 (9)	H10A—C10—H10B	109.5
C10—C7—C8	108.80 (9)	C7—C10—H10C	109.5
C11—C7—C8	110.60 (9)	H10A—C10—H10C	109.5
C6—C7—C8	108.34 (8)	H10B—C10—H10C	109.5
C8A—C8—C7	112.23 (8)	C7—C11—H11A	109.5
C4A—C8A—O1	122.61 (9)	C7—C11—H11B	109.5
C4A—C8A—C8	125.70 (9)	H11A—C11—H11B	109.5
O1—C8A—C8	111.69 (8)	C7—C11—H11C	109.5
N2—C9—C3	178.34 (11)	H11A—C11—H11C	109.5
C13—C12—C21	121.76 (10)	H11B—C11—H11C	109.5
C12—C13—C14	118.75 (10)	C13—C12—H12	119.1
C12—C13—C4	121.77 (9)	C21—C12—H12	119.1
C14—C13—C4	119.37 (9)	C15—C14—H14	119.5
C15—C14—C13	120.90 (11)	C13—C14—H14	119.5
C14—C15—C16	121.22 (11)	C14—C15—H15	119.4
C15—C16—C21	118.42 (10)	C16—C15—H15	119.4
C15—C16—C17	123.10 (12)	C18—C17—H17	119.4
C21—C16—C17	118.48 (13)	C16—C17—H17	119.4
C18—C17—C16	121.23 (14)	C17—C18—H18	119.9
C17—C18—C19	120.17 (12)	C19—C18—H18	119.9
C20—C19—C18	120.53 (14)	C20—C19—H19	119.7
C19—C20—C21	120.63 (13)	C18—C19—H19	119.7
C20—C21—C12	122.14 (11)	C19—C20—H20	119.7
C20—C21—C16	118.93 (11)	C21—C20—H20	119.7
			
C8A—O1—C2—N1	171.82 (8)	C4—C4A—C8A—C8	−172.89 (9)
C8A—O1—C2—C3	−8.36 (13)	C2—O1—C8A—C4A	10.40 (13)
N1—C2—C3—C9	−3.54 (16)	C2—O1—C8A—C8	−170.59 (8)
O1—C2—C3—C9	176.67 (9)	C7—C8—C8A—C4A	19.52 (14)
N1—C2—C3—C4	170.13 (10)	C7—C8—C8A—O1	−159.46 (8)
O1—C2—C3—C4	−9.65 (14)	C21—C12—C13—C14	−0.51 (15)
C2—C3—C4—C4A	22.65 (13)	C21—C12—C13—C4	−176.64 (9)
C9—C3—C4—C4A	−163.67 (8)	C4A—C4—C13—C12	−37.80 (12)
C2—C3—C4—C13	−100.65 (11)	C3—C4—C13—C12	82.98 (11)
C9—C3—C4—C13	73.03 (11)	C4A—C4—C13—C14	146.09 (9)
C3—C4—C4A—C8A	−20.86 (12)	C3—C4—C13—C14	−93.12 (11)
C13—C4—C4A—C8A	101.57 (11)	C12—C13—C14—C15	−0.50 (16)
C3—C4—C4A—C5	160.51 (8)	C4—C13—C14—C15	175.72 (10)
C13—C4—C4A—C5	−77.05 (11)	C13—C14—C15—C16	0.66 (17)
C8A—C4A—C5—O2	178.62 (9)	C14—C15—C16—C21	0.19 (17)
C4—C4A—C5—O2	−2.71 (14)	C14—C15—C16—C17	−179.62 (12)
C8A—C4A—C5—C6	0.61 (14)	C15—C16—C17—C18	−179.19 (13)
C4—C4A—C5—C6	179.28 (8)	C21—C16—C17—C18	0.99 (19)
O2—C5—C6—C7	150.21 (10)	C16—C17—C18—C19	0.3 (2)
C4A—C5—C6—C7	−31.83 (13)	C17—C18—C19—C20	−1.2 (2)
C5—C6—C7—C10	173.48 (9)	C18—C19—C20—C21	0.8 (2)
C5—C6—C7—C11	−66.28 (11)	C19—C20—C21—C12	−179.56 (11)
C5—C6—C7—C8	54.40 (11)	C19—C20—C21—C16	0.47 (17)
C10—C7—C8—C8A	−167.89 (9)	C13—C12—C21—C20	−178.63 (10)
C11—C7—C8—C8A	72.21 (11)	C13—C12—C21—C16	1.34 (15)
C6—C7—C8—C8A	−47.78 (11)	C15—C16—C21—C20	178.82 (10)
C5—C4A—C8A—O1	−175.40 (8)	C17—C16—C21—C20	−1.36 (16)
C4—C4A—C8A—O1	5.99 (15)	C15—C16—C21—C12	−1.16 (16)
C5—C4A—C8A—C8	5.73 (15)	C17—C16—C21—C12	178.66 (10)

### Hydrogen-bond geometry (Å, º)

**Table:**

*D*—H···*A*	*D*—H	H···*A*	*D*···*A*	*D*—H···*A*
N1—H01···N2^i^	0.90 (1)	2.11 (1)	2.9948 (12)	170 (1)
N1—H02···O2^ii^	0.90 (1)	1.94 (1)	2.8404 (11)	176 (1)
C6—H6*B*···N2^iii^	0.99	2.69	3.6366 (14)	160

Symmetry codes: (i) −*x*+1, −*y*+2, −*z*+1; (ii) *x*, −*y*+1, *z*−1/2; (iii) *x*, *y*−1, *z*.
